# Evaluation and validation of *de novo* and hybrid assembly techniques to derive high-quality genome sequences

**DOI:** 10.1093/bioinformatics/btu391

**Published:** 2014-06-14

**Authors:** Sagar M. Utturkar, Dawn M. Klingeman, Miriam L. Land, Christopher W. Schadt, Mitchel J. Doktycz, Dale A. Pelletier, Steven D. Brown

**Affiliations:** ^1^Graduate School of Genome Science and Technology, University of Tennessee, Knoxville, TN 37919, USA and ^2^Biosciences Division, Oak Ridge National Laboratory, Oak Ridge, TN 37831, USA

## Abstract

**Motivation:** To assess the potential of different types of sequence data combined with *de novo* and hybrid assembly approaches to improve existing draft genome sequences.

**Results:** Illumina, 454 and PacBio sequencing technologies were used to generate *de novo* and hybrid genome assemblies for four different bacteria, which were assessed for quality using summary statistics (e.g. number of contigs, N50) and *in silico* evaluation tools. Differences in predictions of multiple copies of rDNA operons for each respective bacterium were evaluated by PCR and Sanger sequencing, and then the validated results were applied as an additional criterion to rank assemblies. In general, assemblies using longer PacBio reads were better able to resolve repetitive regions. In this study, the combination of Illumina and PacBio sequence data assembled through the ALLPATHS-LG algorithm gave the best summary statistics and most accurate rDNA operon number predictions. This study will aid others looking to improve existing draft genome assemblies.

**Availability and implementation:** All assembly tools except CLC Genomics Workbench are freely available under GNU General Public License.

**Contact:** brownsd@ornl.gov

**Supplementary information:** Supplementary data are available at *Bioinformatics* online.

## 1 INTRODUCTION

The development and evolution of next-generation sequencing (NGS) platforms has dramatically changed biological studies in recent years (Mavromatis *et al.*, 2012). Assembly of DNA reads to correctly reconstruct genomes is an essential task to facilitate genomic studies, and a variety of assembly algorithms and methods for quality evaluation have been developed (Nagarajan and Pop, 2013). However, most sequenced genomes are incomplete owing to technical difficulties, time and the expense leading to an increasing disparity in quality and usefulness between finished and draft genomes in databases (Chain *et al.*, 2009).

Because of their low cost, accuracy and high throughput, Illumina platforms have dominated the sequencing industry (Mavromatis *et al.*, 2012). Short read sequencing technologies have limited power to resolve large repetitive regions even within relatively small microbial genomes (Nagarajan and Pop, 2013). The so-called ‘third generation’ single-molecule sequencing technology developed by Pacific Biosciences (PacBio) has been compared with several NGS platforms (Quail *et al.*, 2012). Read lengths up to 14 kb have been reported for PacBio RS I chemistry (Nagarajan and Pop, 2013) and nearly 27 kb for RS II chemistry (Brown *et al.*, 2014).

Repetitive DNA such as ribosomal DNA (rDNA) operons present one of the greatest technical challenges during the assembly process, which is exacerbated when repeat sequence regions are longer than the read lengths (Treangen and Salzberg, 2012). In many cases, where repetitive DNA is present, short read genome assemblies remain highly fragmented and often only achieve high-quality draft status (Chain *et al.*, 2009). The relative value of a finished genome (Fraser *et al.*, 2002), technical challenges (Hurt *et al.*, 2012; Treangen and Salzberg, 2012) and what is missing from finished versus draft quality genomes (Mavromatis *et al.*, 2012) have been discussed previously. Several strategies proposed and implemented for improving genome assemblies include the use of varying size fragment libraries, longer length reads, gap-closure software and postprocessing to detect misassemblies (Treangen and Salzberg, 2012).

Recently, draft genome sequences for 41 bacteria isolated from the *Populus deltoides* rhizosphere and endosphere were obtained using an Illumina Hiseq2000 instrument, and the genomes were represented by 187 contigs, on average (Brown *et al.*, 2012b). An additional two genomes were unsuitable for publication at that time because of high contig numbers, and 10 of the 43 genomes contained >280 contigs. The aim of this study was to compare and select the most appropriate NGS technology combinations, assembly protocol and parameter optimization to improve the genome assemblies of the *Rhizobium* sp. strain CF080 and *Burkholderia* sp. strain BT03 that originally proved problematic, as well as two other strains, *Pseudomonas* sp. strain GM41 and *Pseudomonas* sp. strain GM30 of biological interest. In addition to a variety of *in silico* techniques for evaluation of genome assemblies, a PCR and Sanger sequencing strategy was used to validate rDNA operon predictions and further assess the assemblies.

## 2 METHODS

**DNA sequence data generation:** Illumina paired-end (PE) sequencing has been described (Brown *et al.*, 2012b). Illumina mate-pair (MP) libraries with an average insert size of 6 kb were prepared using the Nextera mate-pair Sample Preparation Kit following the manufacturer’s protocols, and sequencing was completed on a MiSeq instrument. Roche 454 libraries were prepared following the ‘Rapid Library Preparation’ method according to manufacturer’s recommendations for single-end pyrosequencing using the Roche 454 GS FLX System and Titanium XLR70+ kit (Roche 454). PacBio sequencing data were generated at the Genome Sequencing and Analysis Core Resource at Duke University using the PacBio RS-I instrument, C2 chemistry and one SMRT cell per genome. Raw sequence data from all the platforms are available through the NCBI SRA database under accession number SRP010852.

**Sequence data trimming, filtering, annotation and assembly:** Quality trimming and filtering of Illumina reads was performed as described previously (Brown *et al.*, 2012b). The assemblers used for the *de novo* and hybrid assembly, their respective versions and assembly recipes are provided in the Supplementary Information (Section S1). The final assemblies were annotated by the Prodigal gene calling algorithm (Hyatt *et al.*, 2010) and Integrated Microbial Genomes system (Markowitz *et al.*, 2012). The best hybrid assemblies for strain CF080, GM30, BT03 and GM41 were deposited at the NCBI GenBank database under accession numbers AKKC00000000, AKJP00000000, AKKD00000000 and AKJN00000000, respectively.

**Assessment of genome assembly quality and rDNA analysis:** The *in silico* evaluation of assemblies was performed using computing genome assembly likelihoods (CGAL) (version 0.9.6) and REAPR (version 1.0.16) tools, rDNA operon prediction was performed using RNAmmer software (version 2.3.2) and alignments were created using Geneious software (version 6.1.5) (Auckland, New Zealand). PCR amplification and Sanger sequencing protocols are provided (Supplementary Section S1, Supplementary Tables S1 and S2).

## 3 RESULTS AND DISCUSSION

### 3.1 Sequencing details

Illumina PE data were available (Brown *et al.*, 2012b), and additional sequencing was performed using Roche 454, Illumina MP and PacBio RS-I platforms. The average read lengths and coverage values from each sequencing platform are summarized (Table 1). Previously published draft genome assemblies generated from Illumina PE reads (Brown *et al.*, 2012b) were improved using combined data from the different sequencing platforms and hybrid assembly protocols.

**Table 1.** Summary of sequence data coverage

NGS Technology	Illumina PE	Illumina MP	Roche 454 SE	PacBio
Avg. Read Length (bp)	100	150	565	5456
BT03	240x*	24x	15x	18x
CF080	475x	41x	26x	20x
GM41	520x	46x	24x	32x
GM30	520x	36x	26x	NA

*Note:* *x defines raw read coverage value.

A non-hybrid assembly method HGAP has been developed that requires 80–100× of PacBio sequence coverage (Chin *et al.*, 2013), and several recent studies have shown that assembly of PacBio data alone generated the most complete and accurate *de novo* assemblies for several bacteria (Brown *et al.*, 2014; Koren *et al.*, 2013)*.* In this study, *de novo* assembly of PacBio RS I data only with the HGAP method generated poor-quality assemblies (highly fragmented with low N50 values and having smaller genome size than expected), which was likely because of the relatively low sequence coverage (18–32×). Hence, hybrid assemblies for these four strains were compared using summary statistics, assembly evaluation tools and rDNA content. The performance of each hybrid assembly algorithm is described below. However, for new PacBio sequence data generation, one should aim for >100× coverage using the RS II Sequencing System, which can obtain better genome assemblies (Chin *et al.*, 2013).

In a recent example, a closed, high-quality genome sequence for *Clostridium autoethanogenum* DSM10061 was generated using only the latest single-molecule DNA sequencing technology and without the need for manual finishing (Brown *et al.*, 2014). Comparison of the PacBio assembly to assemblies based on shorter read DNA technologies (454, Ion Torrent, and Illumina) showed they were confounded by the large number repeats and their size, which in the case of the rRNA gene operons were ∼5 kb. The *C. autoethanogenum* PacBio sequence data cost ∼US$ 1500. A detailed cost-analysis for different sequence data types has been reported (Koren *et al.*, 2013). Longer reads, greater sequencing depth, the random nature of single molecule sequencing errors and its cost and assembly performance suggests this technology will be increasingly used to produce finished microbial genomes (Koren *et al.*, 2013).

### 3.2 Assembly of data from Illumina PE

The initial assemblies of Illumina PE reads were mostly generated using CLC genomics workbench (CLC) (Brown *et al.*, 2012b). We used the same dataset and alternative assembly algorithms such as Velvet (Zerbino and Birney, 2008), SOAP (Luo *et al.*, 2012), ABySS (Simpson *et al.*, 2009), MaSuRCA (Zimin *et al.*, 2013) and SPAdes (Bankevich *et al.*, 2012), which obtained improved assembly statistics. The SPAdes assembler generated the best summary statistics using Illumina PE reads with an exception of strain CF080. The ABySS assembler performed consistently for all four strains, as it generated similar statistics to the SPAdes assembler as well as generating the best assembly for strain CF080 using PE data. The performance of the MaSuRCA assembler was genome and data dependent, as it generated poor assembly statistics for strain BT03 and GM30 while reasonable assembly statistics for strain CF080 and GM41 (Supplementary Table S3).

### 3.3 Assembly of Illumina PE and MP data

MP libraries are capable of resolving repetitive regions and structural variants while increasing the accuracy and size of assembled contigs (Ribeiro *et al.*, 2012). Short reads could be best assembled through de Bruijn Graph (DBG) assembly approach (Miller *et al.*, 2010). The PE-MP hybrid assemblies generated by DBG-based ABySS, SOAP, Velvet and MaSuRCA were only slightly better than the previously published PE-only assemblies (Brown *et al.*, 2012b), whereas greater improvements in summary statistics were obtained by SPAdes and ALLPATHS-LG assemblers (Table 2). In this study, the ALLPATHS-LG algorithm (Butler *et al.*, 2008) outperformed the SPAdes assemblies in terms of contig numbers and generated superior hybrid assemblies. The optimal performance of ALLPATH-LG can be attributed to a specific type of library requirement where PE and MP reads are designed to overlap each other and can be joined to yield roughly twice the read length of individual reads (Nagarajan and Pop, 2013). In recent years, the ALLPATHS-LG algorithm has arguably won the Assemblathon (Earl *et al.*, 2011) and GAGE (Salzberg *et al.*, 2012) competitions by using this assembly approach.

**Table 2.** Summary of *de novo* and hybrid assembly results

Strain	Library type	No. of contigs	Maximum contig size (kb)	N50 (kb)	Genome size (Mb)	No. of scaffolds	Max Scaffold size (kb)	N50 (kb)	Genome size (Mb)	Software
CF080	PE	1039	335	75	7.54	897	631	383	7.56	CLC
	PE*	90	694	237	8.20	69	646	331	7.20	ABySS
	454	71	1058	236	7.01	—	—	—	—	Newbler
	Pacbio-454	102	799	187	7.06	—	—	—	—	PBcR
	PE-454	57	1225	483	7.02	—	—	—	—	Newbler
	PE-MP	163	1413	597	7.12	103	4100	4100	7.21	MaSuRCA
	PE-MP*	40	1535	626	7.04	12	4813	4813	7.10	ALLPATHSLG
	PE-MP-454	252	4095	4095	7.23	249	4095	4095	7.23	MaSuRCA
	PE-MP-454*	32	1341	615	7.01	—	—	—	—	Newbler
	PE-MP-454-Pacbio	—	—	—	—	6	4102	4102	7.04	AHA
	PE-MP-Pacbio	25	2395	1779	7.04	23	2395	1844	7.04	SPAdes
	**PE-MP-Pacbio**	**16**	**1885**	**671**	**7.04**	**5**	**4797**	**4797**	**7.05**	**ALLPATHSLG**
GM41	PE	164	308	75	6.61	89	599	137	6.64	CLC
	PE*	101	436	165	6.64	96	679	183	6.64	SPAdes
	454	112	236	89	6.61	—	—	—	—	Newbler
	Pacbio-454	80	371	140	6.79	—	—	—	—	PBcR
	PE-454	96	345	143	6.63	—	—	—	—	Newbler
	PE-MP	157	621	279	6.70	117	2057	1560	6.71	MaSuRCA
	PE-MP	86	436	183	6.71	80	681	183	6.72	SPAdes
	PE-MP*	62	415	107	6.65	5	3919	3919	6.72	ALLPATHS-LG
	PE-MP-454	66	345	159	6.62	—	—	—	—	Newbler
	PE-MP-454-Pacbio	—	—	—	—	17	1007	666	6.67	AHA
	PE-MP-Pacbio	73	653	292	6.68	68	1070	292	6.69	SPAdes
	PE-MP-Pacbio*	**13**	**2562**	**1393**	**6.68**	**4**	**2835**	**2408**	**6.68**	**ALLPATHSLG**
GM30	PE	180	184	59	6.14	55	567	227	6.17	CLC
	PE*	61	662	186	6.15	52	662	208	6.16	SPAdes
	454	74	326	133	6.14	—	—	—	—	Newbler
	PE-454	54	801	183	6.15	—	—	—	—	Newbler
	PE-MP	50	661	240	6.20	45	661	333	6.20	SPAdes
	PE-MP*	44	472	229	6.16	4	6208	6208	6.21	ALLPATHSLG
	**PE-MP-454**	**32**	**543**	**298**	**6.15**	**—**	**—**	**—**	**—**	**Newbler**
BT03	PE	690	155	29	10.64	422	295	63	10.77	CLC
	PE*	397	363	80	10.82	386	363	85	10.83	SPAdes
	454	305	344	59	10.75	—	—	—	—	Newbler
	Pacbio-454	235	565	99	11.40	—	—	—	—	PBcR
	PE-454	315	344	70	10.82	—	—	—	—	Newbler
	PE-MP	806	240	59	10.95	457	1997	1161	11.04	MaSuRCA
	PE-MP	362	364	77	11.16	355	364	85	11.17	SPAdes
	PE-MP*	**135**	**562**	**177**	**10.91**	**22**	**2542**	**1282**	**11.11**	**ALLPATHSLG**

*Note:* *Defines the optimal assembly statistics for particular combination of library types as assembled by more than one assembler. The best assembly is shown in bold. The hybrid assembly statistics which were worse than the PE assemblies are not included in above table. The complete table of *de novo* and hybrid assemblies is available through Supplementary Table S3.

### 3.4 Hybrid assembly of Illumina and Roche 454 data

Longer reads from 454 platform could be best assembled through overlap-layout-consensus approach (Miller *et al.*, 2008). The assembly of native, shotgun 454 reads through Newbler generated better summary statistics as compared with PE data alone (Table 2). One 454-Illumina hybrid assembly approach involved merging the 454-only assembly with Illumina reads by PHRAP (version 1.09) (de la Bastide and McCombie, 2007) or Minimus (version 3.0.1) (Sommer *et al.*, 2007) to extend contigs. In this study, PHRAP and Minimus merged assemblies often generated aberrant results (e.g., 1–2 Mb genome assemblies for 5–6 Mb *Pseudomonas* genomes) and contained a high number of singleton (non-assembled) sequences. Additionally, hybrid assembly is supported by the CLC, MaSuRCA and Celera (Miller *et al.*, 2008) assemblers. Hybrid assembly of Illumina and 454 reads was expected to exceed the 454 only assembly statistics based on earlier studies (Brown *et al.*, 2012a). However, CLC did not substantially improve the assembly statistics. MaSuRCA hybrid assemblies with PE-MP-454 combination generated improved N50 values but contained high number of contigs as compared with 454 only assemblies of four strains (Supplementary Table S3).

**Fig. 1.**
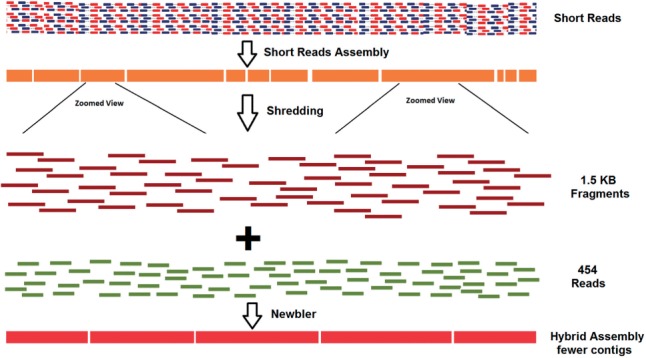
Overview of 454 and Illumina hybrid assembly. Representation of shredding approach to generate 454 and Illumina hybrid assembly

The Newbler software supports fasta/fastq input along with native 454 reads. However, when quality-trimmed Illumina reads or draft assembly of Illumina reads were used as additional input, Newbler failed to complete the assembly process. This was likely because of the large size of Illumina data or long fasta sequences, respectively. Therefore, draft assemblies were cut into 1.5 kb pseudo reads with 300 bp overlap using fb_dice.pl script from the FragBlast module (http://www.clarkfrancis.com/codes/fb_dice.pl) and assembled together with native 454 reads using Newbler (Fig. 1), as described previously (Brown *et al.*, 2012a), which alleviated failure issues and resulted in substantial improvements in N50 statistics, and appropriate genome size estimates were maintained (Table 2). The *in silico* approach to generate 1.5 kb overlapping pseudo reads was influenced by the quality of initial draft assembly. Shredding of PE-MP hybrid assemblies (which had better summary statistics) achieved better results as compared with shredding of PE only assemblies. Therefore, it appears that even when using this shredding technique, generating the optimal draft genome assemblies from Illumina data before the shredding is an important step towards successful hybrid assembly. Any misassembly in the initial assembly risks being propagated into the hybrid assembly.

To attain insight into the draft assembly generation, summary statistics of previously published draft assemblies of 43 bacterial isolates (Brown *et al.*, 2012b) generated using four different assemblers are given (Supplementary Tables S4 and S5), and important parameters that influenced the assembly process are described below. Poor-quality sequencing reads can adversely affect the assembly process (Salzberg *et al.*, 2012), and we observed that quality-based trimming of raw data gave ∼15-fold improvements in N50 statistics. The assembly of PE Illumina reads by the ABySS and SPAdes assembler generated highest N50 statistics when compared with results from the Velvet, SOAP and CLC assemblers (Supplementary Tables S3–S5). Different Kmer values were tested (Chikhi and Medvedev, 2014) and optimal summary statistics were obtained at higher Kmer values, up to 60, and beyond this value summary statistics deteriorated (Supplementary Tables S4 and S5). The increase in raw read coverage up to 300× generated concomitant increases in N50 values, while beyond 300× coverage, the N50 statistics did not increase (Supplementary Fig. S1). Therefore, the quality and sequence coverage of raw reads, Kmer value and appropriate assembly algorithm selection are essential parameters for optimization of draft genome assemblies. We recommend using the ABySS assembler with Illumina PE data and ALLPATHS-LG or SPAdes assembler with Illumina PE-MP data for optimal results. Although we used N50 statistics for the initial short listing of assemblies, it should be noted that large N50 values are not always indicative of assembly quality, and additional validation should be performed using various bioinformatics tools as described by (Koren *et al.*, 2014) and rDNA analysis approach described below.

### 3.5 Hybrid assembly of Illumina, 454 and PacBio data

Single molecule sequencing technology currently produces the longest read lengths across all NGS platforms, and the performance of PacBio RS sequencing system has been compared with other NGS platforms recently (Liu *et al.*, 2012; Quail *et al.*, 2012). The longer reads generated with the PacBio system have the potential to exceed the longest repeats in most bacterial genomes and greatly improve the genome assemblies (Koren *et al.*, 2013). However, PacBio sequencing technology has a high error rate, which has been reported as being 18% (Nagarajan and Pop, 2013). Different hybrid assembly protocols have been developed to overcome the high error rates associated with the single molecule sequencing technology and limitations of short-read technologies (Bashir *et al.*, 2012; English *et al.*, 2012; Koren *et al.*, 2012; Ribeiro *et al.*, 2012). Various hybrid assembly protocols to improve earlier assemblies were pursued and results are described below.

#### 3.5.1 PacBio corrected Reads (PBcR) pipeline

The higher error rate associated with PacBio technology obscures the read alignments and complicates the assembly process. Most genome assemblers are unable to handle this high error rate, and hence error correction becomes necessary to unlock the full potential of longer reads for *de novo* assembly. The PBcR pipeline uses higher fidelity Illumina and/or 454 reads to trim and correct the individual long-read sequences and generates hybrid consensus with >99.99% base-call accuracy (Koren *et al.*, 2012). We used 454 reads to correct errors in PacBio reads through the PBcR pipeline, which were then assembled via the Celera assembler (Miller *et al.*, 2008). The PBcR hybrid assembly statistics were similar to those generated with PE-MP and PE-454 combinations (Table 2). The PBcR assemblies contained few collapsed repeats as compared with other assemblies (Supplementary Table S6), which is likely a product of longer, corrected reads. It should be noted that like HGAP, the PBcR pipeline is also capable of performing self-correction and non-hybrid assembly of PacBio reads when sufficient (∼100×) coverage is available. However, because of the PacBio coverage limitation we could not perform the self-correction approach.

#### 3.5.2 The AHA scaffolding method

The AHA scaffolding approach (Bashir *et al.*, 2012) is available through the SMRT analysis package (version 2.0, Pacific Biosciences), and it uses any previous assembly to which longer PacBio reads are aligned using the BLASR algorithm (Chaisson and Tesler, 2012) to create higher, ordered scaffolds. We used the best contig assembly generated through PE-MP-454 combination and error corrected PacBio reads as an input to AHA protocol. The resulting scaffolds were ranked second best after the ALLPATHS-LG (Table 2).

#### 3.5.3 ALLPATHS-LG

The ALLPATHS-LG recipe uses a mixture of three data types, where Illumina PE and MP reads are assembled first using DBG approach, and then PacBio reads are incorporated to patch coverage gaps and resolve repeats (Maccallum *et al.*, 2009). The ALLPATHS-LG method requires all inputs in raw format and uses its own error correction pipeline. ALLPATHS-LG assemblies with PE-MP combination were found to be superior to the numerous other protocols compared here and consistent with earlier studies (Earl *et al.*, 2011; Salzberg *et al.*, 2012). Incorporation of PacBio reads with this method further improved the assembly results up to ‘noncontiguous finished’ quality (Table 2). However, incorporation of PacBio reads was memory intensive, the software crashed multiple times on a high memory (132 GB) server, and it was unable to assemble the BT03 genome. This behaviour may be attributed to some combination of computational memory limitation; higher genome BT03 size (∼11 Mb); and its content (the genome contained numerous phage and transposon sequences). Our datasets contained one MP library with ∼6 kb insert sizes and achieved near-finished genome assemblies. Ribeiro *et al.* used multiple MP libraries with insert sizes ranging from 2–6 kb and were able to generate finished or near-finished assemblies for different bacterial genomes (Ribeiro *et al.*, 2012). Hence, inclusion of multiple MP libraries of varying length could be a possible path to further improve the assemblies in the future.

#### 3.5.4 SPAdes

Recent GAGE-B comparisons identified SPAdes as one of the best algorithms for bacterial genome assemblies using Illumina data. Consistent with previous findings, SPAdes performed well to assemble our four genomes using Illumina PE-MP data. Recently SPAdes added support for the PacBio data, which allowed a direct comparison of its performance with ALLPATHS-LG for PE-MP-PacBio combinations. The overall summary statistics generated by both assemblers were similar but ALLPATHS-LG assemblies always contained lower contig numbers than SPAdes. Notably, SPAdes seamlessly assembled the PE-MP-PacBio combination for strain BT03 for which ALLPATHS-LG encountered crashing issues associated with memory limitation.

#### 3.5.5 Gap-filling by PBJelly algorithm

The PBJelly method (English *et al.*, 2012) aligns PacBio/454 reads to the scaffold assembly to extend the contigs and resolve the gaps. The PBJelly algorithm was applied to the best scaffolded assemblies generated by ALLPATHS-LG together with the PacBio reads. PBJelly was able to fill up (64, 99 and 93%) gaps in BT03, CF080 and GM41 genomes, respectively (Table 3). Many microbial genomics analyses depend on the finished genomes and single unbroken contig is important for a wide range of disciplines (Koren *et al.*, 2013). Scaffolded assemblies are helpful in the genome finishing process and are used to determine contig order and contig overlap (Nagarajan *et al.*, 2010; Swain *et al.*, 2012). Long range PacBio reads offer an attractive opportunity to reduce the number of gaps and resolve unidentified base-pairs (N’s) in the scaffolds, which reduces the overall cost of manual finishing.

**Table 3.** Summary of PBJelly gap-filling results

Description		BT03	CF080	GM41
^a^Input assembly statistics	Number of Gaps	96	7	5
	Total Gap Length (bp)	195,912	2,880	3,475
^b^PBJelly assembly statistics	Number of Gaps	26	2	3
	Total Gap Length (bp)	70,100	30	232

*Note:* ^a^Gap statistics for the best scaffold assembly. ^b^Gap statistics after application of PBJelly algorithm.

### 3.6 Assembly quality assessments and comparisons

Although the assembly metrics such as N50 and contig numbers are widely used for the assembly evaluation, they may not always correlate well with the actual quality of the assembly (Nagarajan and Pop, 2013) and several other bioinformatics approaches and metrics have been developed to assess assembly quality (Gurevich *et al.*, 2013; Hunt *et al.*, 2013; Koren *et al.*, 2014; Rahman and Pachter, 2013). The CGAL is one recent approach that incorporates genome coverage and assembly accuracy into the evaluation without need of reference sequence and combines them into a single metric score (Rahman and Pachter, 2013). The CGAL software ranked the SPAdes assemblies as highest, while ALLPATHS-LG and MaSuRCA assemblies have scores close to the SPAdes assemblies (Supplementary Table S7). The REAPR genome assembly evaluation tool generates a positional error call metric, assesses potential collapsed repeats and single base-by-base scores (Hunt *et al.*, 2013). The REAPR evaluation generated the least number of error calls for the ALLPATHS-LG assemblies generated with Illumina only (PE-MP) data (Supplementary Table S6). CGAL and REAPR both assigned high rankings to ALLPATHS-LG assemblies likely reflecting their higher accuracy and depth of coverage.

On the other hand, hybrid assemblies using 454/PacBio reads that had better summary statistics were assigned with lower CGAL scores and a large number of error calls by REAPR (Supplementary Tables S6 and S7). These inconsistent scores by CGAL/REAPR are possibly because of the design limitation of these *in silico* evaluation tools, which cannot currently use 454/PacBio reads during the evaluation. The 454/PacBio reads may have included data for repetitive regions that are not spanned by the Illumina reads and reported as errors based on evaluation by Illumina reads. To improve the consensus accuracy of PacBio assemblies, we performed assembly polishing using the Quiver tool (Chin *et al.*, 2013). However, low coverage of PacBio reads may not have achieved the required base-call quality and contributing toward low scores by *in silico* evaluation tools. REAPR detected fewer collapsed repeats in the assemblies using PacBio reads (Supplementary Table S6), and this suggests that the longer PacBio reads better resolved repetitive regions.

Reciprocal blastp analyses were conducted using proteins predicted from the draft and the best hybrid assemblies to gain insights into potential protein encoding differences (Table 4). The majority (87–98%) of proteins were unchanged by assembly improvements supporting the notion that for some studies draft quality genome sequences may be sufficient. However, a substantial number of proteins were longer after assembly improvement, and a number of new proteins were predicted in most cases. The majority of newly predicted proteins were for hypothetical proteins, and others included genes with predicted regulatory functions or metabolic genes such as for a putative nitric oxide dioxygenase. The number of potential missing genes will be genome and assembly-specific, and this is difficult to assess in the absence of available finished reference genomes (Fraser *et al.*, 2002).

**Table 4.** Comparison of Open Reading Frames (ORFs) predicted in draft and improved genome assemblies

Strains	CF080	BT03	GM30	GM41
^a^Total ORFs	6684	10 056	5511	5975
^b^No. of unchanged ORFs	5819	9385	5424	5881
No. of longer ORFs	786	413	77	71
No. of shorter ORFs	64	205	10	15
No. of new ORFs	15	53	0	8

*Note:* ^a^Total number of open reading frames predicted in improved genome assembly by Prodigal gene calling algorithm. ^b^Number of open reading frames in improved genome assemblies as compared with draft assemblies.

### 3.7 Assembly validation

The CGAL and REAPR evaluation methods were only able to rank the assemblies based on number of errors, and verification of the error calls would require finished reference genome sequences, which were beyond the scope of the present study. Therefore an additional level of verification was necessary to better assess assembly accuracy. As genome assemblers are often confounded by large repetitive regions (e.g. 5–7 kb rDNA operons), (Treangen and Salzberg, 2012) accurate prediction of rDNA operon was selected as an additional criterion to assess the assembly accuracy and to gain insight into potential systematic issues.

Several copies of 5S, 16S, and 23S rDNA elements were predicted for strains CF080, GM41, GM30 and BT03, and in this study, the complete rDNA operon is defined as an arrangement of 5S, 16S, and 23S rDNA elements in single operon structure on a single contig. rDNA genes were predicted by the RNAmmer program (Lagesen *et al.*, 2007) and predictions were tested using a PCR-based approach. Briefly, oligonuclelotides were designed to bind to DNA regions that were 5′ and 3′ to the predicted rDNA operons and give amplified products of a predicted size. Additional internal oligonucleotides were designed to amplify and sequence end regions. Correct assembly of the rDNA operon was expected to generate a PCR product in the desired size range, while an incorrectly assembled rDNA operon would fail to amplify or give unexpected sequence lengths. Measured and expected product sizes for positive PCR reactions for each rDNA operon in each strain are shown (Supplementary Table S1), along with the length of DNA sequence that was verified by Sanger sequencing (Supplementary Table S2). These presumptive positive results support this experimental approach, although the entire PCR product could be sequenced by primer-walking for increased assembly confidence.

#### 3.7.1 rDNA operons in Rhizobium sp. strain CF080

**Fig. 2.**
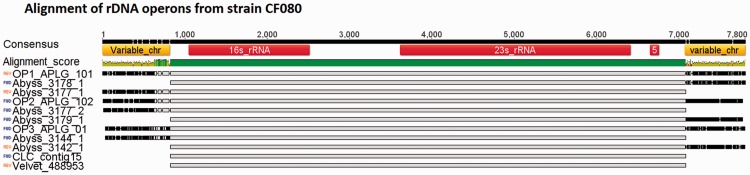
Alignment of predicted CF080 rDNA operons tested via PCR and Sanger sequencing. The names of the operon denote corresponding assembly algorithm (ALLPATHS-LG is displayed as APLG) and contig ID. The alignment mismatches are highlighted in black and matches in grey. Identity of overlapping sequences is shown on top of the alignment as colored bar; positions with 100% identity are in green and positions with lower identity are in yellow. The annotation and the genomic position are shown on the consensus sequence

Summary statistics and bioinformatics assessment suggested the ALLPATHS-LG assembly was optimal for strain CF080 (Table 2, Supplementary Tables S4, S6 and S7) and three rDNA operons, and their flanking chromosomal regions were predicted on three separate contigs (Fig. 2). The SPAdes assembly with PE-MP-PacBio combination have also predicted three rDNA operons and similar arrangement as in ALLPATHS-LG assemblies. Three copies of rDNA operons have been detected within six finished *Rhizobium* genomes sequences. The ∼7 Mb ALLPATHS-LG genome assembly supported predictions for three rDNA operons that were validated by PCR and Sanger sequencing. ABySS generated an assembly that was ∼8 Mb in size and it supported predictions for six rDNA operon copies (Fig. 2). However, the ABySS assembly was unable to resolve regions of DNA that were 5′ and 3′ of different rDNA operons leading to their duplication within the assembly (Fig. 2). The rDNA operon duplication in the ABySS assembly accounts for a portion but not all of the higher genome size reported. Previous studies that used the ABySS assembly method have also noted that ABySS assembler predicted larger genome sizes as compared with other methods (Haridas *et al.*, 2011; Salzberg *et al.*, 2012) but did not identify the specific reasons for these higher genome sizes. The Velvet and CLC algorithms were able to assemble only one complete rDNA operon in strain CF080 and unable to predict flanking chromosomal regions; this is likely a contributing factor to these assemblies being more fragmented (Table 2). Hence, the ALLPATHS-LG assembly having the best summary statistics and accurate prediction of three copies of rDNA operons was selected as the best assembly for strain CF080. An analysis of rDNA operons in *Pseudomonas* sp. strains GM41 and GM30, and in *Burkholderia* sp. strain BT03 are presented (Supplemental Fig. S2).

### 3.8 Comparison of assembly approaches

In this study, we examined a variety of *de novo* genome assembly methodologies for four novel bacterial isolates that do not have existing reference sequences. There are a large number of different assemblers and different parameters that one can use for *de novo* studies. Numerous recent studies report continued assembly developments and comparisons, which reflects the importance of generating a high-quality, representative genome sequence (Bradnam *et al.*, 2013; Powers *et al.*, 2013). It has been shown that a number of assemblers perform well when a single metric is considered but few perform consistently across a set of quality metrics. In this study, in addition to a range of *in silico* methods, we experimentally examined rDNA operons predictions from different assemblies, which provided an additional criterion for assembly quality assessment.

## 4 CONCLUSIONS

The ABySS and SPAdes software generated the best assembly statistics when only PE Illumina reads were used. ABySS assembler performed well consistently for all four genomes and also correctly identified multiple copies of rDNA operons (Fig. 2, Supplementary Fig. S2). As expected, additional sequencing data from each NGS platform improved the assembly statistics (Table 2). Hybrid assemblies with PE-MP data combinations were superior as compared with PE-454 combinations. However, the superiority of the PE-MP combination can likely be attributed to the excellent performance of the ALLPATHS-LG and SPAdes algorithms. Inclusion of PacBio data resulted in substantial improvements in assembly statistics but success was dependent on the selection of assembly approach. The PBcR assembly statistics were comparable with that of the PE-454 combination. The AHA and PBJelly methods facilitated scaffolding and gap-filling, respectively and would be helpful during genome finishing. Among the 11 *de novo* and hybrid assembly protocols tested here, the ALLPATHS-LG assembler with the combination of PE-MP-PacBio data generated the best results and also provided the most accurate rDNA operons predictions, except in the case of the BT03 genome, where computational resource limitations prevented evaluation. These results underscore the importance of comparing multiple appropriate algorithms and key parameters for genome assembly. Our results were consistent with earlier studies that demonstrated the advantage of including longer PacBio reads (Roberts *et al.*, 2013; Shin *et al.*, 2013) and our hybrid assembly results with PacBio data demonstrate the power of these longer reads to better resolve repetitive sequence regions. The evaluation framework described here should prove useful for others looking to improve existing draft genome sequences.

Our results showed that by using complementary libraries, sequencing technologies and appropriate hybrid assembly protocols, dramatic improvements in assembly quality for bacterial genomes could be obtained. The rDNA operon analysis through PCR and Sanger sequencing provided additional confidence for the assembly accuracy. The genomes for strains GM41 and GM30 were previously defined as ‘high-quality draft’ (Brown *et al.*, 2012b) using described criteria (Chain *et al.*, 2009), while previous assemblies for CF080 and BT03 consisted of 1039 and 690 contigs, respectively. The improved CF080 and BT03 genomes are now represented by 16 and 135 contigs, respectively. CF080 and GM41 assemblies can now be termed as ‘noncontiguous finished’, where automated improvements have been performed and most of the gaps have been resolved (5 and 4 scaffolds, respectively). The GM30 and BT03 can be termed as ‘improved high-quality draft’.

## Supplementary Material

Supplementary Data
