# Supplementary material for: Evaluation and validation of de novo and hybrid assembly techniques to derive high-quality genome sequences
Source: Bioinformatics. 2014 Jun 14;30(19):2709–16. doi: 10.1093/bioinformatics/btu391 (PMC4173024; doi:10.1093/bioinformatics/btu391)
Supplement: Supplementary Data [file supp_30_19_2709__index.html]

Evaluation and validation of de novo and hybrid assembly techniques to derive high quality genome sequences — Evaluation and validation of de novo and hybrid assembly techniques to derive high-quality genome sequences — Evaluation and validation of de novo and hybrid assembly techniques to derive high-quality genome sequences — Supplementary Data 

# Evaluation and validation of *de novo* and hybrid assembly techniques to derive high-quality genome sequences

## Supplementary Data

files

**Files in this Data Supplement:**

- Supplementary Data - docx file
- Supplementary Data - doc file
- Supplementary Data - xlsx file
