# Supplementary material for: Evaluation and validation of de novo and hybrid assembly techniques to derive high-quality genome sequences
Source: Bioinformatics. 2014 Jun 14;30(19):2709–16. doi: 10.1093/bioinformatics/btu391 (PMC4173024; doi:10.1093/bioinformatics/btu391)
Supplement: Supplementary Data [file supp_btu391_Supplemental_Figure.docx]

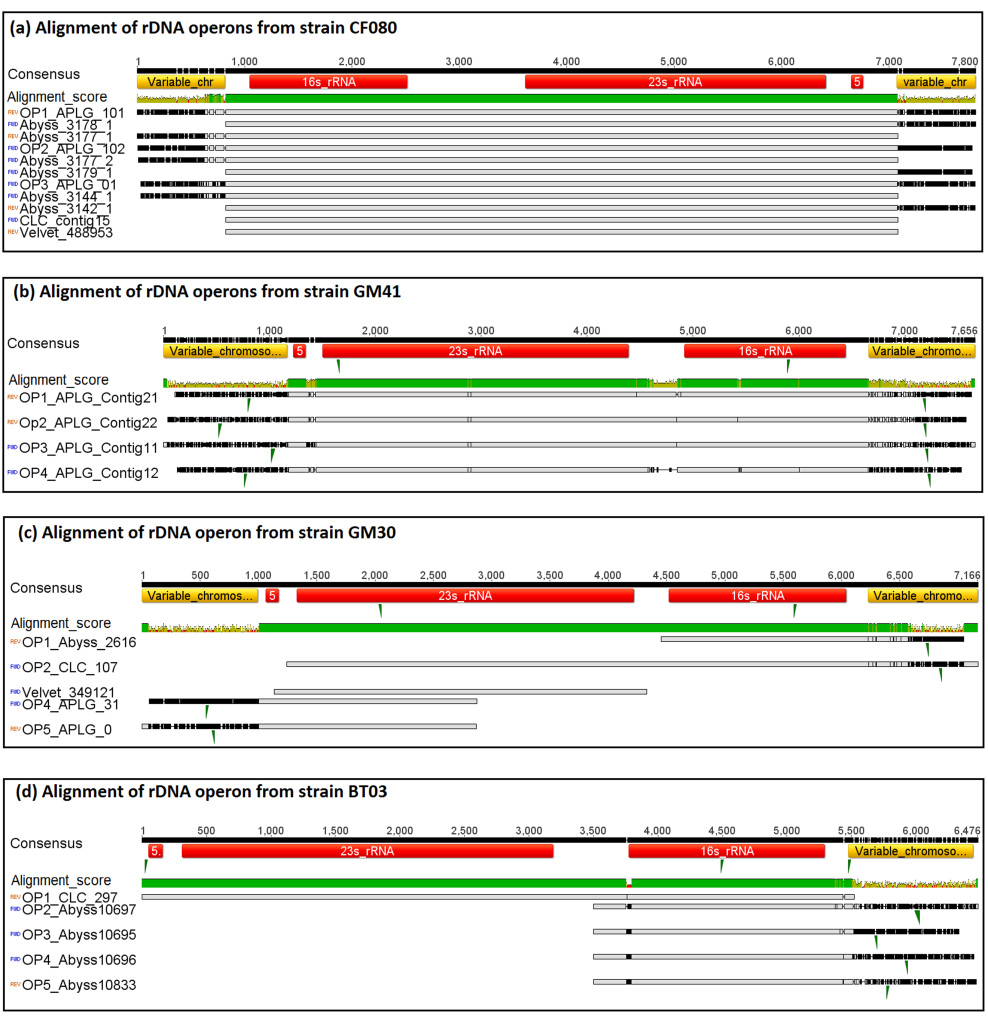
**Supplemental Fig.** Alignment of predicted rDNA operons tested via PCR and Sanger sequencing. Alignment of predicted rDNA operons in strain (b) GM41 (c) GM30 (d) BT03 is shown. Multiple copies of rDNA operon were detected by variability in the alignment identity and designated with prefix ‘OP’. The names of the operon denotes corresponding assembly algorithm (ALLPATHS-LG is displayed as APLG) and contig ID. The annotation and the genomic position are shown on the consensus sequence. The PCR primer sequences are denoted as green triangles and alignment identity is shown as “Alignment_score”. * The “Alignment_score” correspond to the color (Green – 100%, Red – Below 50%) and height of the graphic.

*rDNA operons in Pseudomonas sp. strain GM41*

Two to seven rDNA operons have been detected within 35 finished *Pseudomonas* genomes sequences available through IMG database ([Markowitz, et al., 2012](#_ENREF_34)). For strain GM41, all four copies of rDNA operons from the ALLPATHS-LG assembly were verified by PCR and Sanger sequencing (b). The ALLPATHS-LG hybrid assembly predicted all four copies on separate contigs with their corresponding flanking chromosomal regions. SPAdes assembly predicted a one complete rDNA operon along with its flanking chromosomal regions. The CLC assembly was able to assemble a single complete rDNA operon but flanking chromosomal regions and multiple copies were missing. Velvet and ABySS were only able to assemble the individual (5S, 16S, and 23S) rDNA elements but the rDNA operon structure was incomplete. Hence for strain GM41 the ALLPATHS-LG assembly predicted multiple copies of the rDNA operons, which were supported by the PCR and Sanger sequencing, thus providing additional confidence in its quality.

*rDNA operons in Pseudomonas sp. strain GM30*

The ABySS, Velvet, SPAdes and CLC assemblies each supported predictions for only one partial rDNA operon (containing any two of the 5S, 16S, or 23S rDNA sequences) while the ALLPATHS-LG assembly predicted two partial rDNA operon copies (c). PCR and Sanger sequencing assessments identified four partial rDNA operons, that each had unique associated flanking DNA. Based on the operon arrangement it was hypothesized that contigs, labelled OP5_APLG_0 and OP1_Abyss_2616 could be joined, and tests based upon PCR and Sanger sequencing were able to join these contigs into one contiguous DNA sequence. However, a similar PCR strategy was unable to join the contigs labelled OP4_APLG_31 and OP2_CLC_107 (c). Hence, we were able to merge two partial operons into one complete rDNA operon. The poor assembly of complete rDNA operons in strain GM30 was attributed to a lack of PacBio data. This strain was characterized by the production of surfactant-like compound that may have interfered with the PacBio chemistry as sequencing failed in two attempts.

*rDNA operons in Burkholderia sp. strain BT03*

There are two to seven rDNA operons in 35 *Burkholderia* genomes available through IMG database ([Markowitz, et al., 2012](#_ENREF_34)) and assemblies for strain BT03 supported one complete and four partial rDNA operons. One complete operon was identified by CLC and SPAdes while the remaining four were partial operons (identified by ABySS) (d). All five operons were confirmed by PCR and Sanger sequencing. The complete operon from CLC/SPAdes does not include the flanking chromosomal region while ABySS predicted operons include only 3’ flanking chromosomal regions. It is possible that operon found in CLC/SPAdes assembly is the same as the one predicted from ABySS assembly. However, the exact arrangement could not be validated as 5’ flanking chromosomal regions were missing from all our rDNA operon assemblies. The four copies of partial rDNA operons could prove useful if future manual finishing is to be undertaken. As mentioned previously, PacBio reads were unable to be incorporated into the ALLPATHS-LG assembly due to computational resource limitations.
