# Supplementary material for: Evaluation and validation of de novo and hybrid assembly techniques to derive high-quality genome sequences
Source: Bioinformatics. 2014 Jun 14;30(19):2709–16. doi: 10.1093/bioinformatics/btu391 (PMC4173024; doi:10.1093/bioinformatics/btu391)
Supplement: Supplementary Data [file supp_btu391_Supplemental_revised_1_4-16-2014_updated_Sagar.doc]

**Section S1. Supplementary methods:**

**Genome Assembly**

Software versions:

ABySS (version 1.3.2), Velvet (version 1.2.1), CLC Genomics Workbench (version 4.7), SOAPdenovo (version 1.05), ALLPATHS-LG (release 44849), Newbler (version 2.6), PHRAP (version 1.09), Minimus (version 3.0.1), PBJelly (version 12.9.14), PBcR pipeline (version 7.0), AHA and HGAP – SMRTanalysis (version 2.0), SPAdes (version 3.0.0), MaSuRCA (version 2.2.1)

Assembly Recipe:

The genome assembly recipes for Velvet, ABySS and SOAPdenovo and ALLPATHS-LG were followed from the GAGE protocols . The genome assembly recipes for Spades and MaSuRCA were followed from the respective user manuals. The CLC Genomics Workbench and SMRT analysis assemblies were performed using default settings. The hybrid assemblies with Newbler were generated as described previously .The PHRAP, Minimus, PBJelly and PBcR assemblies were performed as per the instructions in respective manuals and with default parameters.

**Assessment of genome assembly quality and rDNA analysis**

Assembly evaluation

The summary statistics for the assembly were calculated using (summrizeAssembly.py) script which is part of PBJelly software. The CGAL (version 0.9.6) and REAPR (version 1.0.16) assembly evaluations were performed as per the instructions in the respective manuals and with default parameters.

Prediction of rDNA oeprons

Individual rDNA (16S, 23S and 5S) sequences were predicted using RNAmmer software (version 2.3.2) and operon arrangements were determined manually using genomic positions. The 5’ and 3’ flanking chromosomal region of rDNA operon (1,000 bp on either side) were extracted (when available) using the custom Perl script. Alignments of rDNA sequences (including 5’ and 3’ flanking chromosomal regions) were performed using Geneious software (version 6.1.5) (Auckland, New Zealand).

PCR and Sanger sequencing

The PCR primers for each predicted rDNA operon were designed using Primer3 software and PCR reactions were carried out using Phusion high-fidelity PCR master mix with HF buffer (New England BioLabs) according to the manufacturer’s instructions. The PCR amplification conditions include annealing for 30 seconds (at temperature stated in Table S1), an extension at 72^0^ C for 1 minute (for products <2 kb) or 10 minute (for products < 2 kb) with 20 cycles. The PCR product purification was performed using QIAquick PCR purification kit as per the manufacturer’s instruction. The verification of PCR products was performed through Sanger sequencing by employing standard approach described previously .


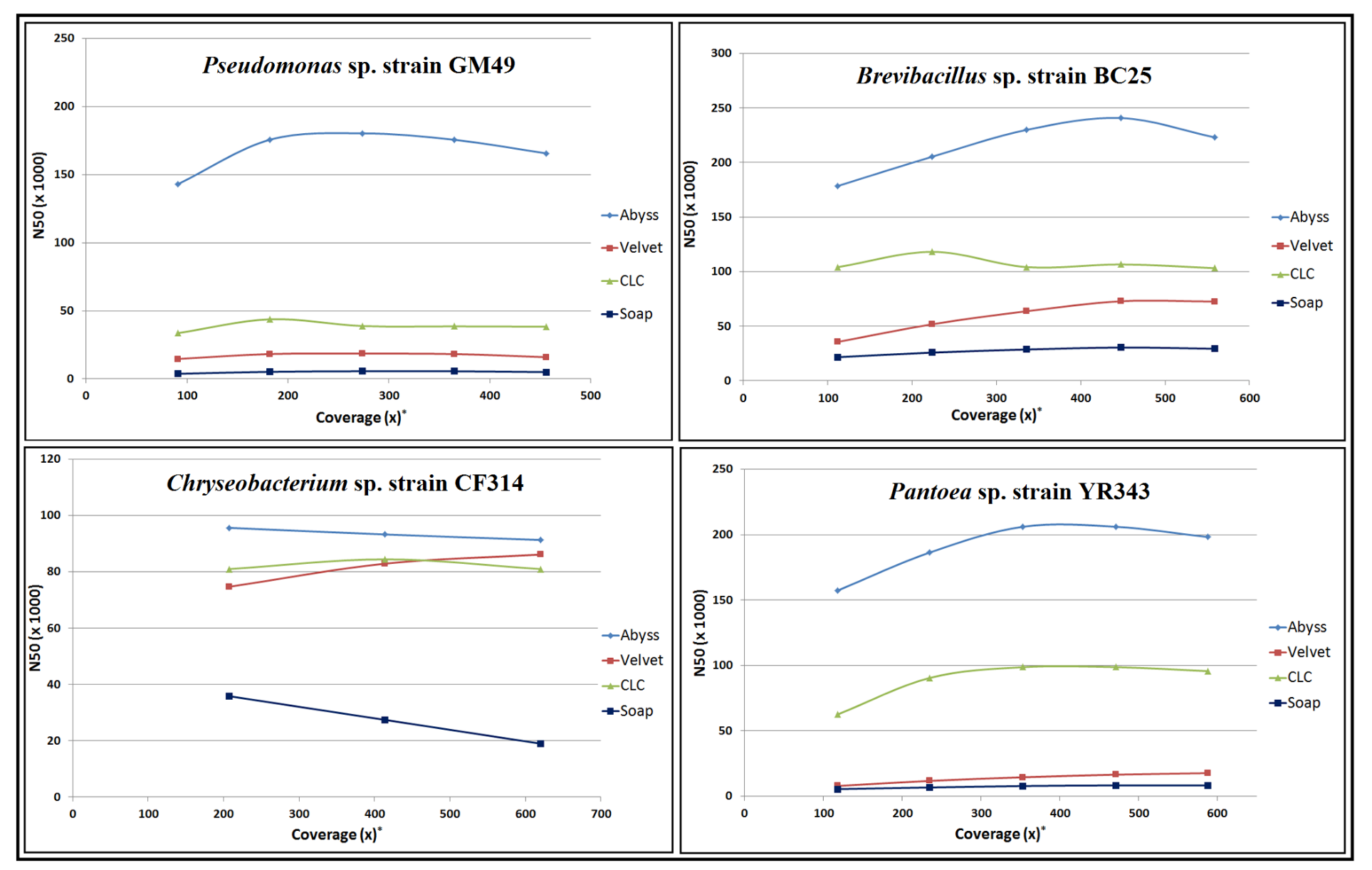
**Figure S1**. Coverage analysis. The genome assemblies of four isolates were created at incremental raw read coverage levels using ABySS, Velvet, CLC and SOAP software. The optimal N50 values generated by each software at various coverage levels are shown.

**References:**

Brown, S.D.*, et al.* (2011) Mutant alcohol dehydrogenase leads to improved ethanol tolerance in *Clostridium thermocellum*, *Proc Natl Acad Sci U S A*, **108**, 13752-13757.

Brown, S.D.*, et al.* (2012) Draft genome sequence of *Rhizobium* sp. strain PDO1-076, a bacterium isolated from *Populus deltoides*, *J. Bacteriol.*, **194**, 2383-2384.

Lagesen, K.*, et al.* (2007) RNAmmer: consistent and rapid annotation of ribosomal RNA genes, *Nucleic Acids Res.*, **35**, 3100-3108.

Salzberg, S.L.*, et al.* (2012) GAGE: A critical evaluation of genome assemblies and assembly algorithms, *Genome Res.*, **22**, 557-567.

Untergasser, A.*, et al.* (2012) Primer3--new capabilities and interfaces, *Nucleic Acids Res.*, **40**, e115.
